# An Exonuclease III Protection-Based Electrochemical Method for Estrogen Receptor Assay

**DOI:** 10.3390/ijms140510298

**Published:** 2013-05-16

**Authors:** Sha Zhu, Ya Cao, Yuanyuan Xu, Yongmei Yin, Genxi Li

**Affiliations:** 1 Department of Oncology, the First Affiliated Hospital of Nanjing Medical University, Nanjing 210029, China; E-Mail: nanyizhusha@126.com; 2 Department of Biochemistry and State Key Laboratory of Pharmaceutical Biotechnology, Nanjing University, Nanjing 210093, China; E-Mail: xuyuanyuan@njau.edu.cn; 3 Laboratory of Biosensing Technology, School of Life Sciences, Shanghai University, Shanghai 200444, China; E-Mail: conezimint@shu.edu.cn

**Keywords:** estrogen receptor, estrogen response elements, Exonuclease III, electrochemical method

## Abstract

Estrogen receptor (ER), expressed in approximately 80% of primary breast cancer cells, has proven to be a valuable predictive factor of the disease. Herein, by making use of the specific binding of ER to its DNA response elements, we propose an Exonuclease III (Exo III) protection-based electrochemical method for detecting ER proteins. In this assay, the presence of ER can protect the duplex DNA molecules immobilized on an electrode surface from Exo III-catalyzed digestion, resulting in an increased electrochemical signal. Experimental results have revealed that the proposed method can allow the quantification of ER in the range of 0.5 to 100 nM with a satisfactory detection limit of 0.38 nM. Furthermore, since this approach can also be employed to detect ER directly in nuclear extracts, it may be of great use in biomedical applications in the future.

## 1. Introduction

Estrogen receptor (ER), a ligand-dependent transcription factor, is a member of the nuclear hormone receptor superfamily [1,2]. The biological effects of ER are mediated by its binding to estrogen, and are then involved in activating transcription either by direct binding to its own DNA response elements (estrogen response elements, EREs) or by tethering to other transcription factors [3,4]. Emerging findings suggest that ER plays an important role in the biology of breast cancer [5,6]. As a result, ER has been regarded as a valuable marker or target for the diagnosis of breast cancer, and the study to develop new methods for ER assay has attracted increasing interest so as to monitor the related pathological or therapeutical phenomena.

Traditional methods for the detection of ER includes enzyme-linked immunosorbent assay (ELISA) [7], western blotting [8] and immunohistochemistry [9]. Nevertheless, these methods usually present some shortcomings. For example, immunohistochemistry and western blotting are typically employed in a semiquantitative mode [10,11], while ELISA can produce quantitative data, but is time-consuming and expensive because of the requirement of specific labeled antibodies. Recently, Lee and coworkers developed a novel metal nanoparticle-based colorimetric assay to quantitatively detect ER by utilizing the specific ER-ERE interaction [12]. The method involves two sets of double-stranded (ds) DNA modified nanoparticles, each carrying a half site segment of EREs with a short complementary sticky end. The presence of ER will promote annealing of the complementary ends and stabilize the full ERE structure, generating detectable signals. This assay is rapid, accurate and low-cost; but is still restricted by the poor detection limit (25 nM), which is attributed to the fact that the ER binding affinity may decrease by the split of the EREs.

In this paper, we report a novel electrochemical method to detect ER, which can also be used as a tool to monitor ER status in cells. The biosensing strategy is based on the protection of DNA duplex from Exonuclease III (Exo III)-mediated digestion by specific binding of ER to EREs. With the merits of simplicity, quick response, high sensitivity, easy operation and low cost, the proposed method can overcome disadvantages of traditional methods, and may have potential for further clinical applications.

## 2. Results and Discussion

**Scheme 1:**
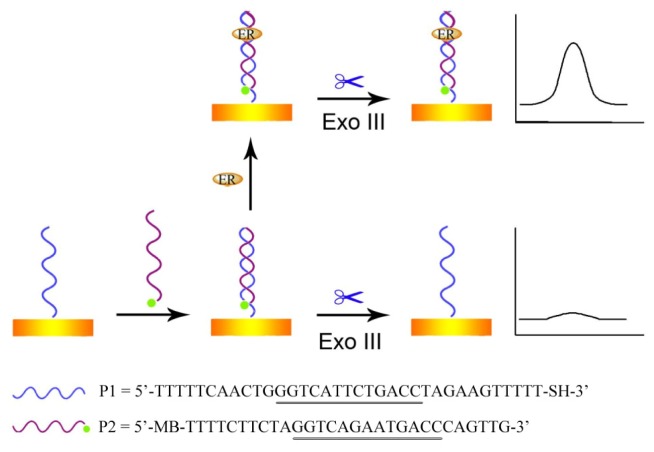
Schematic illustration of the mechanism to develop an electrochemical method for sensitive detection of estrogen receptor (ER). The underlined sequences represent the estrogen response elements (EREs).

The principle of the developed electrochemical biosensor for sensitive detection of ER has been illustrated in Scheme 1. First, two complementary DNA strands incorporating the 5′-GGTCAnnnTGACC (n = spacer nucleotides)-3′ EREs [13,14] are prepared. Among them, P1 is thiolated to ensure self-assembly on the gold electrode surface, while P2 is modified with methylene blue (MB) to serve as an electroactive signaling probe. Hybridization of P1 and P2 grafts the P2 strand to be immobilized on the electrode surface, forming a stable duplex with a 3′-blunt end at the signaling strand. Then, Exo III, which has a high exodeoxyribonuclease activity for duplex DNAs in the 3′ to 5′ direction [15–21], recognizes the 3′-blunt end and catalyzes the stepwise removal of mononucleotides from 3′-hydroxyl termini of P2, thus the signal tag MB is removed leading to a low electrochemical response. However, if there is ER in the test system, the digestion of the exonuclease will be blocked by the formation of ER-DNA complex due to the significant steric hindrance. Since increased ER will protect more P2 from digestion, it results in an increased electrochemical signal. The relationship between the ER concentration and the obtained electrochemical signal can then be established, which forms the basis of the new assay method for the detection of ER.

**Figure 1:**
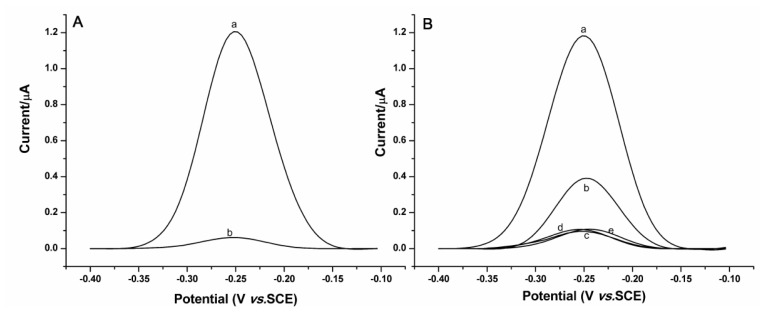
Square wave voltammograms obtained at the gold electrode immobilized with the DNA strands. (**A**) In the absence of ER, (a) before, and (b) after the DNA strands are digested by Exo III; (**B**) In the presence of 100 nM ER, (a) before, and (b) after the DNA strands are digested by Exo III. Curves c–e are for the control experiments by using 500 nM bovine serum albumin (BSA), thrombin and α-fetoprotein (AFP) instead of 100 nM ER. Buffer: 10 mM phosphate-buffered saline (PBS) buffer (PH 7.4).

To demonstrate the utility of our design, we first investigated the digestion reaction of the P1-P2 duplex by Exo III. As shown in Figure 1A, square wave voltammetry (SWV) response of the free duplex demonstrates an oxidation peak contributed to MB at around −250 mV (curve a); however, after being treated with Exo III, the MB signal completely disappears (curve b), revealing that the enzyme can effectively degrade the duplex and remove the electroactive label. Exo III-mediated digestion of the DNA strands is then performed in the presence of ER, with the results shown in Figure 1B. As is predicted, the sequence-dependent protein-DNA complex formation (ER-ERE) may protect P1-P2 duplex from Exo III digestion, resulting in an observed electrochemical signal (Figure 1B, curve b). So, comparing curve b in Figure 1B with curve b in Figure 1A, we can see that formation of ER-ERE has indeed protected P1-P2 duplex from Exo III digestion and electrochemical signal in relation to ER can be obtained. Therefore an assay of ER by using this design is possible. Furthermore, if irrelevant proteins, such as bovine serum albumin (BSA), thrombin and α-fetoprotein (AFP), which have no specific binding with the ERE, are employed, almost no electrochemical signals can be observed (Figure 1B, curves c–e), although the amount of irrelevant proteins used is much higher than for ER, revealing a good specificity of the assay method.

**Figure 2:**
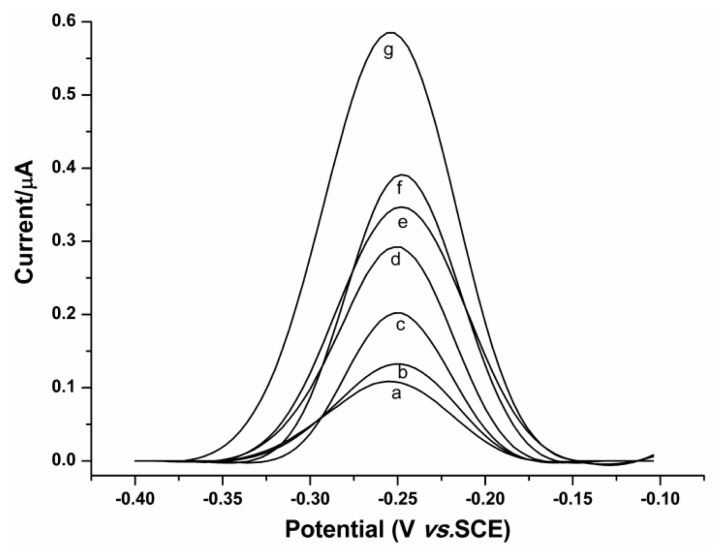
Square wave voltammograms for the measurements of ER with different concentrations: (a) 0.5, (b) 5, (c) 25, (d) 50, (e) 75, (f) 100, (g) 250 nM. Conditions are as in Figure 1.

**Figure 3:**
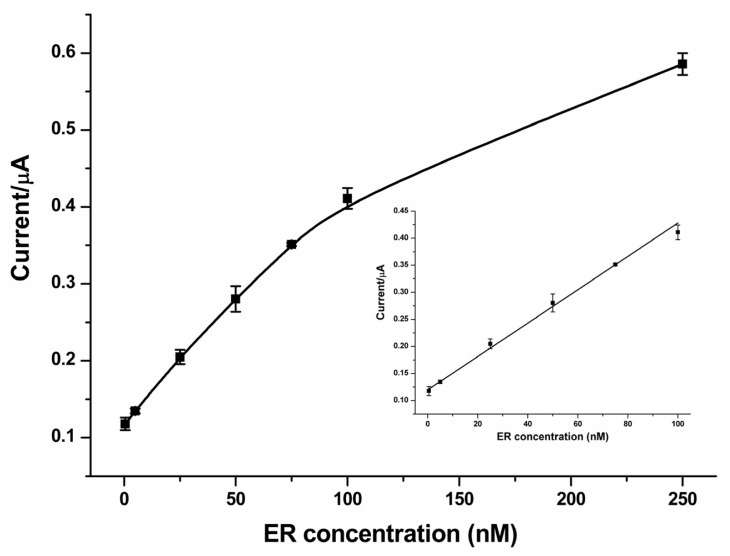
The resulting calibration curve for the electrochemical detection of ER. Error bars represent standard deviations of the measurements (*n* = 3). Inset shows the linear relationship between the square wave voltammetry (SWV) peak current and the ER concentration.

The feasibility of the new method for a quantitative assay of ER has also been examined. Figure 2 displays the typical SWV responses obtained on analyzing ER at different concentrations. It can be observed that the SWV peaks show a gradual increase with increasing ER concentration. This suggests that more MB-labeled P2 molecules were protected from Exo III-catalyzed digestion in the presence of ER of higher concentration. In addition, a linear correlation of the peak currents *versus* the ER concentrations can be obtained in the range from 0.5 to 100 nM (Figure 3, insert). The linear fitting equation was *y* = 0.11964 + 0.00308*x*, where *y* is the peak current (μA); *x* is the concentration of ER (nM); *r* = 0.9956. For each concentration of ER, the measurement has been repeated at least three times independently. The average relative standard deviation (RSD) is 3.92%, revealing that the developed method can be used for quantitative assay of ER with desirable reproducibility. The detection limit is calculated to be 0.38 nM by the interpolation of the mean plus three times the standard deviation of the zero standards [22]; while the limit of quantification (LOQ), which is evaluated as the lowest concentration of ER that can be quantified with acceptable precision and accuracy [23], is experimentally found to be 0.5 nM. Notably, the detection limit of the proposed method had improved by 65-fold as compared with the previously reported ERE-metal nanoparticle-based colorimetric assay (25 nM) [12]. Such significant improvement might be attributed to the avoidance of ERE split and the low background signal. Moreover, the combination of the electrochemical method and the fine-needle aspiration biopsy that can be used to obtain tissue specimens of 5 mg allows an accurate detection of as low as 10 fmol/mg ER in tissues. Because the average level of ER in breast cancer is about 37 fmol/mg [24], the linear range and LOQ of the proposed method can be acceptable for clinical applications.

**Figure 4:**
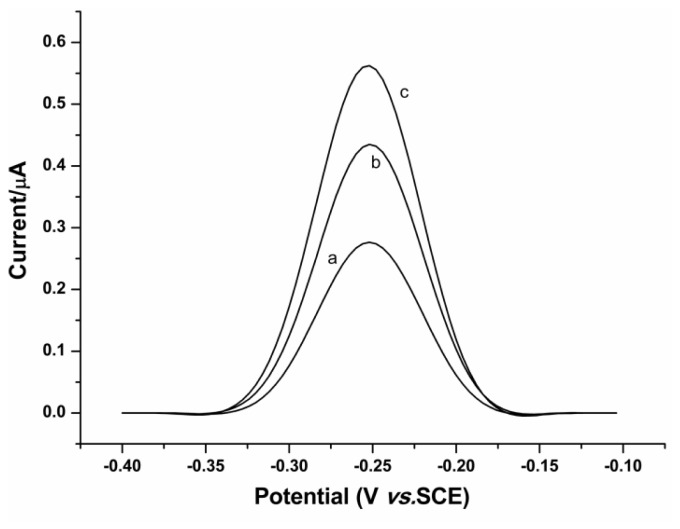
Square wave voltammograms for the assay of ER in nuclear extracts from (a) 2.5 × 10^4^, (b) 5 × 10^4^, (c) 7.5 × 10^4^ MCF-7 cells. Conditions are as in Figure 1.

Given that ER expression level in breast cancer cells is a valuable biomarker in the prediction of ER-targeted therapy, it is important to assess the applicability of the proposed new method for ER detection in nuclear extracts. To this end, an assay of ER from increased amounts of nuclear extracts of MCF-7 cells (2.5 × 10^4^, 5 × 10^4^, 7.5 × 10^4^ MCF-7 cells) has been conducted. The nuclear extracts were prepared by use of a nuclear extract kit (Active Motif, Carlsbad, CA, USA) according to manufacturer’s instructions. As shown in Figure 4, obvious SWV responses can be obtained, consistent with the expression of ER in MCF-7 cell line [25]. Moreover, the SWV peaks increase along with the number of cells, indicating the capability of the method for monitoring ER level in cells.

## 3. Experimental Section

### 3.1. Reagents and Materials

Human recombinant ER (ERα, catalog number: P2187) was purchased from Invitrogen (Carlsbad, CA, USA). Exo III and NEB buffer I was obtained from New England Biolabs (Ipswich, WA, USA). BSA was from Dingguo Biotechnology Co. (Beijing, China). Dithiothreitol (DTT), tris(2-carboxyethyl)phosphine hydrochloride (TCEP), thrombin, AFP and mercaptohexanol (MCH) were from Sigma Aldrich Chemical Co. (Shanghai, China). All other chemicals were of analytical grade and used as received. Oligonucleotides P1 and P2 were synthesized by Takara Biotechnology Co, Ltd. (Dalian, China). Their sequences are listed in Scheme 1.

The buffer solutions used in this work are as follows. DNA immobilization buffer: 10 mM Tris-HCl, 1 mM EDTA, 10 mM TCEP, and 0.1 M NaCl (pH 7.4). Hybridization buffer: 10 mM phosphate-buffered saline (PBS, pH 7.4) with 1 M NaCl. ER binding buffer: 10 mM Tris-HCl, 0.1 mM EDTA, 0.1 mM DTT, 1% glycerol and 200 mM KCl (pH 7.5). All the buffers were prepared with double-distilled water, which was purified with a Milli-Q purification system (Barnstead, MA, USA) to a specific resistance of 18 MΩ cm.

### 3.2. Self-Assembly and Hybridization of the DNA Strands on Gold Electrode Surface

Thiol-modified P1 was firstly self-assembled onto a gold electrode surface via gold–thiol chemistry [26]. Before immobilization of P1 on the electrode surface, the substrate gold electrode was firstly cleaned with freshly prepared piranha solution (70% concentrated sulfuric acid, 30% H_2_O_2_) for 5 min. Then, the electrode was polished on a microcloth with alumina powder of 1, 0.3, and 0.05 μm in sequence. Residual alumina powder was removed by sonicating the electrode sequentially in both ethanol and double-distilled water. After that, the electrode was soaked in nitric acid (50%) for 30 min and scanned in 0.5 M H_2_SO_4_ to remove any remaining impurities. After being dried with purified nitrogen, the electrode was immediately used for P1 immobilization by incubation of the electrode with 0.2 μM P1 for 16 h. To remove non-specific DNA adsorption and block the remaining nonspecific binding sites, the gold electrode was further treated with 1 mM MCH solution for 30 min and then rinsed with double-distilled water. Hybridization was conducted at 37 °C by immersing the above prepared electrode in 1 μM MB-labeled P2 for 2 h. After hybridization, the electrode was thoroughly rinsed with double-distilled water and used for the following experiments.

### 3.3. Electrochemical Detection of ER

Electrochemical detection of ER was performed at 37 °C as follows: Recombinant ER was incubated with the above prepared electrode in 100 μL of ER binding buffer for 30 min. Then, the electrode was thoroughly rinsed and immersed in a 100 μL of 1× NEB buffer I that contains 2 units ExoIII. The ExoIII digestion was sustained for 1 min and terminated by adding 20 mM EDTA. Afterward, the electrode was rinsed thoroughly with double-distilled water and used for the following measurements.

### 3.4. Electrochemical Measurements

Electrochemical measurements were carried out on a Model 660C Electrochemical Analyzer (CH Instruments, Shanghai, China) with a conventional three-electrode system at room temperature. The three-electrode system consisted of the modified electrode as the working electrode, a saturated calomel electrode (SCE) as the reference electrode, and a platinum wire as the counter electrode. The experiments were performed in 10 mM PBS buffer (PH 7.4) using square wave voltammetry (SWV) with a 40 mV amplitude signal at a frequency of 90 Hz, over the potential range from −0.4 to −0.1 V.

## 4. Conclusions

In summary, we have designed an electrochemical biosensor as a new tool for ER detection. The assay combines the specific ER–ERE interaction with an ExoIII protection-based strategy, achieving a detection limit of 0.38 nM, which represents a sensitivity improvement of up to 65-fold compared with the previously reported colorimetric assay. Moreover, the feasibility of using the method directly in cell nuclear extracts has also been demonstrated. Since clinical ER status in breast tumors is a valuable prognostic indicator and predictive factor for response to hormonal therapy, this new method may help ongoing research in improving the effectiveness of therapeutic interventions for breast cancer in the future.
